# Deconstructing synthetic biology across scales: a conceptual approach for training synthetic biologists

**DOI:** 10.1038/s41467-024-49626-x

**Published:** 2024-06-26

**Authors:** Ashty S. Karim, Dylan M. Brown, Chloé M. Archuleta, Sharisse Grannan, Ludmilla Aristilde, Yogesh Goyal, Josh N. Leonard, Niall M. Mangan, Arthur Prindle, Gabriel J. Rocklin, Keith J. Tyo, Laurie Zoloth, Michael C. Jewett, Susanna Calkins, Neha P. Kamat, Danielle Tullman-Ercek, Julius B. Lucks

**Affiliations:** 1 Center for Synthetic Biology, Northwestern University, Evanston, IL 60208 USA; 2 Department of Chemical and Biological Engineering, Northwestern University, Evanston, IL 60208 USA; 3 Independent Evaluator, Lake Geneva, WI 53147 USA; 4 Department of Civil and Environmental Engineering, Northwestern University, Evanston, IL 60208 USA; 5 Department of Cell and Developmental Biology, Northwestern University, Chicago, IL 60611 USA; 6 Robert H. Lurie Comprehensive Cancer Center, Northwestern University Feinberg School of Medicine, Chicago, IL 60611 USA; 7 Department of Engineering Sciences and Applied Mathematics, Northwestern University, Evanston, IL 60201 USA; 8 Department of Biochemistry and Molecular Genetics, Northwestern University, Chicago, IL 60611 USA; 9 Department of Pharmacology, Northwestern University, Chicago, IL 60611 USA; 10 The Divinity School, University of Chicago, Chicago, IL 60637 USA; 11 Searle Center for Advancing Learning and Teaching, Northwestern University, Evanston, IL 60208 USA; 12 Biomedical Engineering Northwestern University, Evanston, IL 60208 USA; 13 Present Address: Department of Bioengineering, Stanford University, Stanford, CA 94305 USA; 14 Present Address: Nexus for Faculty Success, Rosalind Franklin University of Medicine and Science, Chicago, IL USA

**Keywords:** Synthetic biology, Education

## Abstract

Synthetic biology allows us to reuse, repurpose, and reconfigure biological systems to address society’s most pressing challenges. Developing biotechnologies in this way requires integrating concepts across disciplines, posing challenges to educating students with diverse expertise. We created a framework for synthetic biology training that deconstructs biotechnologies across scales—molecular, circuit/network, cell/cell-free systems, biological communities, and societal—giving students a holistic toolkit to integrate cross-disciplinary concepts towards responsible innovation of successful biotechnologies. We present this framework, lessons learned, and inclusive teaching materials to allow its adaption to train the next generation of synthetic biologists.

## Abstract (web summary)

Developing biotechnologies to address society’s challenges requires integrating concepts across disciplines, posing challenges to educating students with diverse expertise. In this Perspective the authors create a framework for synthetic biology training that deconstructs biotechnologies across spatial scales.

## Introduction

Synthetic biology is the fundamental science and engineering research that allows us to reuse, repurpose, and reconfigure biological systems to address society’s most pressing challenges. Synthetic biologists leverage tools and concepts from biology, chemistry, physics, mathematics, engineering, computer science, and the social sciences to harness the enormous diversity of biological function, creating new biological systems that are advancing agriculture^1,2^, sustainable biomanufacturing^3–5^, and medicine^6–9^. Recognition of this potential has led to synthetic biology becoming a major driver of the growing bioeconomy^10–12^. This in turn has created a surge of interest in synthetic biology, attracting an increasing number of researchers and students from around the world who bring diverse backgrounds and perspectives to the field.

While the potential of synthetic biology is clear, developing an approach to train students that meets the diverse needs of this field faces two related challenges. The first challenge is that the field has developed from threads rooted in multiple individual disciplines, resulting in a broad diversity of concepts that must be taught and integrated. At the core are the biological concepts that explain how a function is encoded within a DNA sequence, how control of gene expression activates this function, and how this function can be changed by manipulating the DNA sequence. Building upon this, early synthetic biology incorporated concepts from physics and computer science abstractions that viewed biological components as being ‘wired’ in genetic networks that controlled information flow, much like electronic circuits^13–15^. At the same time, systems biologists were using some of these concepts to study and manipulate cellular networks and signaling pathways^16,17^, and chemical engineers were using principles of dynamics and control to engineer metabolic processes for bioproduction^18,19^. From these roots, mathematical approaches developed in systems biology were added^20^ as well as concepts from chemistry to create new components not yet found in nature^21^. As the field has advanced concepts out of the lab and into the world, approaches from ethics, social sciences, business, and law have become important to incorporate so that researchers innovate responsibly with positive societal impacts^22–24^.

The conceptual breadth of synthetic biology is difficult to cover in any single training program which gives rise to the second challenge for training in synthetic biology—undergraduate and graduate students are often siloed within single disciplines and degree programs, creating barriers to learning outside of these traditional boundaries. Thus, students receive most of their exposure to synthetic biology through elective courses or research in labs rather than through a structured curriculum as might be associated with other mature disciplines. This can lead to synthetic biology training that emphasizes a narrow set of concepts over others or focuses on content rather than “science practices”^25^ that are known to support deep learning^26,27^. For example, there might be an intense focus on training students how to manipulate CRISPR genome editing systems on the molecular scale, but very little integration of how deficiencies of the molecular-level genome targeting affect the function of the larger cellular system, tissue, or organism in which the CRISPR system is utilized.

The field must overcome these training challenges, as integration of these multi-disciplinary concepts is critical for developing successful synthetic biology technologies. For example, cellular synthesis of products from sustainable feedstocks requires understanding the underlying reaction chemical kinetics (chemistry), enzyme biophysics and substrate transport (physics), genetic regulation of enzymes and cellular physiology (biology), reactor vessel scale-up (engineering), and socio-techno-economic analyzes (business). Similar combinations of expertise are also required to create synthetic biology technologies that address other important societal goals in sustainability, environmental health, and human health.

Fortunately, important first steps to developing new training approaches are beginning to happen with the emergence of new undergraduate opportunities and PhD programs in synthetic biology. For high school students and undergraduates, experiential learning opportunities have emerged to facilitate hands-on learning, such as BioBits Kits^28–31^, the ODIN marketplace for genetic engineering supplies^32^, BioBuilder^33^, and others^34,35^. In addition, opportunities such as the international Genetically Engineered Machines (iGEM) competition, the Build-a-Genome Course^36^, and the Cold Spring Harbor Summer Course in Synthetic Biology have paved the way to explore synthetic biology and this integration of disciplines. Though, there is an opportunity to refine and expand these efforts with an overarching framework that more systematically incorporates concepts from the many fields contributing to synthetic biology. At the PhD level, two notable programs in the US (Rice University) and the UK (Imperial College) have begun to explore systematic approaches to training in synthetic biology. Rice’s PhD program covers physical biology, systems biology, and synthetic biology, requiring one dedicated course in synthetic biology. Imperial College’s program starts with a Master of Research degree followed by a PhD with courses in systems biology and synthetic biology. Both programs are structured to provide training to students to integrate concepts across disciplines but require significant prerequisites in STEM. But how do students who may not have access to one of these programs receive this type of synthetic biology training? The Engineering Biology Research Consortium (EBRC) has worked to address this by creating an “Introduction to Engineering Biology” curriculum module to give students a basic understanding of the tools, technologies, and opportunities in synthetic biology^37^. While each of these programs are important first steps, a critical opportunity remains for creating a new approach to synthetic biology training that can: (1) teach synthetic biologists of the future how to traverse and integrate multiple disciplines into their understanding of the field, no matter what their specific background; (2) be accessible to students from a range of backgrounds in order to democratize opportunity and access to synthetic biology concepts; and (3) be adaptable to incorporate advances in a rapidly changing field.

**Fig. 1:**
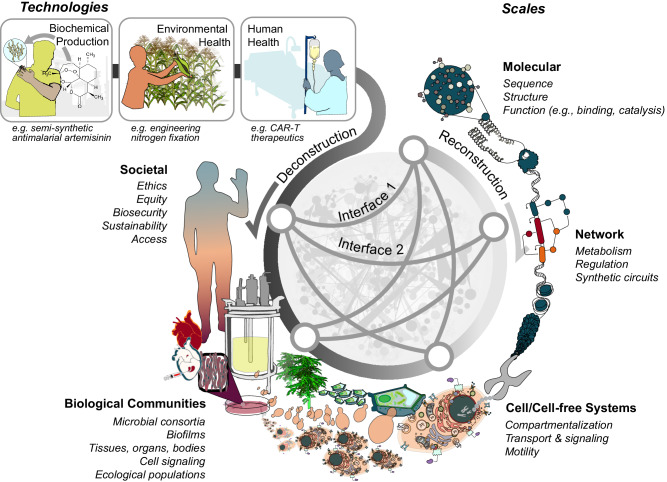
Deconstructing synthetic biology technologies across scales. A schematic representation of the deconstruction framework: biotechnologies can be deconstructed along scales to identify biological phenomena that are important to the technology at each scale, understand the principles by which these phenomena work at that scale, and identify the important interfaces between scales where engineering challenges often arise. Deconstructing technologies along scales allows multidisciplinary concepts to be mapped and applied at individual scales (annotated) and allows new technologies to be reconstructed by combining elements and applying concepts at each scale.

To address this opportunity, we created a conceptual framework for synthetic biology training that can be used in any course or program, developed over the past several years as part of the National Science Foundation-sponsored “Synthesizing Biology Across Scales“ graduate training program at Northwestern University. The framework is based on the observation that every synthetic biology technology is made up of components that function across multiple scales—molecular, circuit/network, cell/cell-free systems, biological communities, and societal—and that the success of these technologies is deeply dependent on their interfaces (Fig. 1). This scales framework can be found in other engineering disciplines as well, such as in electrical and computer engineering where technologies naturally break down along scales, from transistors, to circuits, to chips, to devices, and integrate across scales to enable powerful applications.

Here, we describe a course-based implementation of the scales framework that teaches undergraduates, masters, and PhD students how to deconstruct synthetic biology across scales, analyze how components interact at interfaces between scales to yield emergent phenomena, conceptualize how to combine components across scales to create new synthetic biology solutions to global challenges, and incorporate the consideration of ethics when developing synthetic biology technologies. Our vision is that training students to deconstruct synthetic biology technologies across scales will help them (1) recognize where their domain expertise fits within a particular synthetic biology technology, (2) identify their own knowledge gaps that can be filled through additional topical learning or research collaborations, and (3) gain a holistic picture of the landscape of pieces that must work together to create a successful technology. Each of these “science practices,” which allow students to actively engage in scientific inquiry, promotes disciplinary learning and development as a scientist^25^. Emphasizing the societal scale, we hope to drive responsible innovation by training students to think of concepts in ethics, access, equity, and societal-level impact early and often throughout the development of synthetic biology technologies. We envision that the scales framework and the corresponding deconstruction approach is a launching point for the field of synthetic biology to provide a foundational way of training the next generation of synthetic biologists.

## The scales framework for synthetic biology

The scales framework is a conceptual way to understand how to build synthetic biology solutions to address societal challenges how biological phenomena work across multiple scales (Fig. 1). The deconstruction approach to teaching this framework posits that for a given synthetic biology technology, the components and functions that work together to form that technology can be thought of as working along distinct scales: molecular, circuit/network, cellular, biological communities, and societal. Each of these scales represent a distinct set of components and functions and the physical, chemical, biological, and social science concepts that naturally drive function or impact at that scale. In addition, interactions between components at the interfaces between these scales often give rise to emergent behavior and engineering challenges that are important for real-world applications. Below we briefly describe the components, functions, and concepts that arise at each scale.

The molecular scale includes the individual molecular components of biological systems (e.g., nucleic acids, proteins, lipids, metabolites) and the physical, chemical, and mathematical principles required for understanding and engineering the function of these components. Driving concepts at the molecular scale include the biophysics of protein and RNA folding (including concepts such as free energy folding landscapes and folding kinetics), molecular interactions, enzymology, and others^38,39^. The functions that occur on this scale are molecular structure, complex assembly^40^, catalysis (enzymes)^41^, motion (molecular motors)^42^, charge transport^43^, and others that are carried out by individual molecules.

The components of the network/circuit scale consist of collections of molecules that interact to give rise to higher-order functions, often depending on which subset of interactions are present. Network/circuit scale functions are those that biological systems utilize to propagate information, coordinate physiological states, and implement control over those states^44,45^. Common biological functions at this scale include coordination and regulation of gene expression (transcription/translation), propagation of information in biological systems in signaling networks, and control of molecular transformations in metabolic reaction networks^44–48^.

Phenomena at the cell/cell-free systems scale encapsulate the components of the molecular and network/circuit scale, creating a biochemical environment that supports systems-level functions. Biological functions at this scale include coupled transcription, translation, and post-translational modification^49^, mechanobiology^50^, cell division^51^, exo- and endocytosis^52^, cell sensing^53^, somatic hypermutation (i.e., antibody production)^54^, homeostasis^55^, and transport^55,56^. Sometimes these functions can be spatially organized within a range of cellular components such as lipid vesicles, bacterial microcompartments, and macromolecular condensates that organize molecules in membrane-less organelles^57,58^. At this scale, concepts that govern the behavior include molecular transport, reaction diffusion, energy and redox balance, and others. Cell-free systems are included here because they can perform many of the same functions as cells with similar levels of biological complexity^7,59,60^.

The components of the biological communities scale include multi-cellular interactions and communities of organisms that work together to give rise to higher-order functions and emergent behaviors^61^. There is a rich diversity of systems at this scale, ranging from microbiomes and biofilms to tissues, organs, and even whole bodies^62–66^. Biological functions that occur on this scale include emergent microbial community dynamics^65^, cell-cell signaling^67,68^, biofilm formation^69^, tissue-scale phenomena such as tissue growth^70^, regeneration and function, cell-material interactions, inter- and intraspecies metabolic interaction^71^, and others^72–74^. Population dynamics, microbial ecology, metagenomics, and micro- and macroevolution play a significant role at this scale.

Finally, the societal scale encompasses concepts that will determine how synthetic biology technologies impact, influence, and change the world around us. Functions at this scale include technology distribution; equity and affordability in technology access; social, biological, and economic sustainability; public perception; legal and regulatory aspects of technology (intellectual property and policy); and more^24,75–77^. The concepts associated with this scale include the philosophical ethics of synthetic biology research, stakeholder interaction and analysis, frameworks for user studies and field trials, lifecycle analysis, and quantitative estimates of the needs and viability of synthetic biology technologies^78,79^. Traditionally this scale has been separated from science and engineering at the other scales, yet it contains components and functions driven by scientific principles similar to the other scales. Recognizing the need to train ethically minded practitioners, we emphasize the integration of the societal scale as one of the five key scales so that we consider it as an important part throughout training and technology development.

The interfaces between these scales give rise to emergent behavior important for applications, though this can also present challenges for engineering. By understanding these interfaces, we can learn general “rules” to emergence of complexity and, in turn, engineer-improved technologies. We can understand these interfaces through common methods for bridging across scales. For instance, mathematical techniques such as mean-field averaging, which assumes that many identical components interact in similar ways^80^, and asymptotic analysis, which characterizes the strongest interactions between heterogeneous components^81^, enable us to analyze the transition between scales. The fundamental properties, process, and results of mapping interactions to macro-level behavior inform our understanding of the emergence of complexity across scales^82–86^.

For some technologies that we deconstruct, the scales are clear. Practitioners can identify a global challenge (e.g., chemical production, environmental health, human health) and deconstruct synthetic biology technologies that address them (e.g., semi-synthetic artemisinin, bacterial nitrogen fixation, CAR-T therapeutics) (Box 1). However, for some technologies, scales with strong interfaces may naturally blur together; for instance, it is hard to define exactly when a molecular scale complex that regulates protein phosphorylation begins to process and propagate information through a phosphorylation cascade at the network scale^87^. Learning to deconstruct synthetic biology solutions allows practitioners to understand when the boundaries between scales become ‘fuzzy’, so that they can take advantage of the gradation of phenomena that occur across different spatial and temporal scales and engineer them accordingly. By using case studies on real-world synthetic biology technologies, we can teach core concepts of the field to students from diverse backgrounds in an interactive and engaging way.

Box 1. Deconstruction case studies ▓
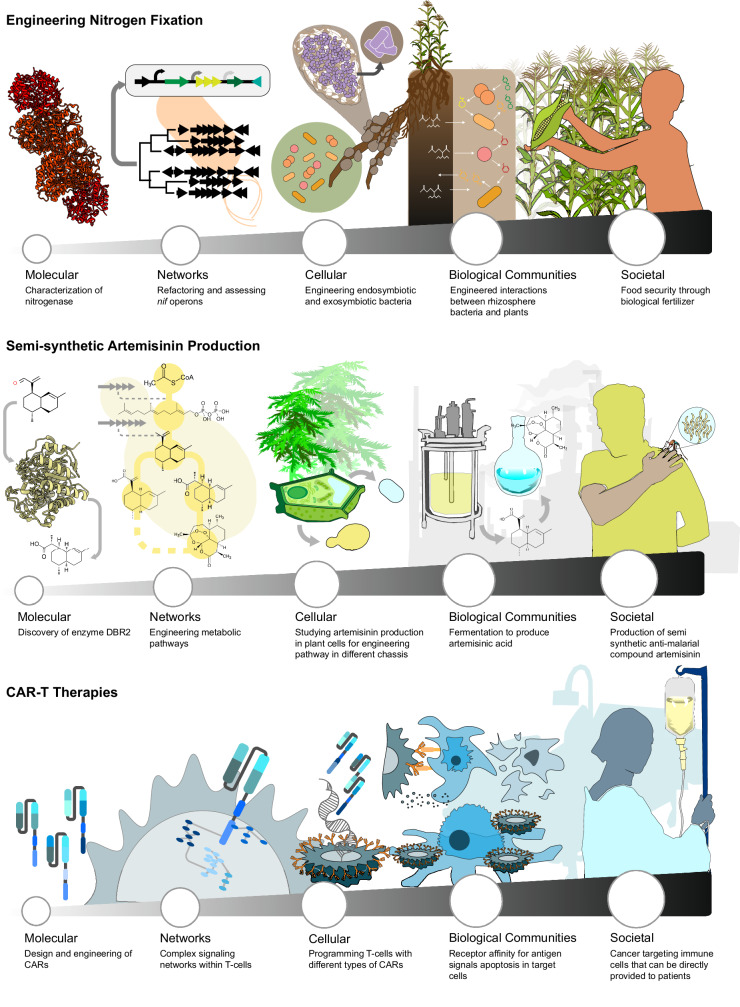

**The deconstruction approach provides a framework to analyze synthetic biology technologies through case studies**. Synthetic biological systems that address challenges in (A) the environment (nitrogen fixation), (B) sustainable bioproduction (semi-synthetic artemisinin production), and (C) human health (CAR-T therapies) are deconstructed along scales.

**Box** 1**Text**. Many synthetic biology technologies can be broken down into components that must work together across the molecular, circuit/network, cellular, and biological communities scales. For each technology, societal scale concepts concerning ethics, equity, access, intellectual property, and business considerations are critical to its success. Here are several examples of flagship synthetic biology technologies deconstructed across these scales.

Environmental Health—nitrogen-fixing bacteria for sustainable fertilizers. Nitrogen-fixing bacteria that can produce fertilizer compounds offer a potential solution for sustainable farming, currently challenged by an over-reliance on energy-intensive chemical fertilizers that cause environmental contamination when overapplied^91^. Engineering a bacteria to produce enough fixed nitrogen for farming needs requires understanding and engineering across scales. At the molecular scale, the core nitrogen-fixing reaction is carried out by the nitrogenase enzyme complex^92,93^. Nitrogenase requires coordinated interaction with electron-transporting proteins that work together at the network/circuit scale^92,108^. Also important at the network/circuit scale are the layers of genetic circuitry that coordinate the synthesis of the many nitrogenase components and its cofactor synthesis enzymes—this regulation must be understood as it presents potential barriers to controlling nitrogenase expression^108^. Both of these scales are embedded in a cellular chassis that must support their function^94,95^. Finally, the eventual application of a nitrogen-fixing bacteria in the soil requires considerations at the biological communities scale to understand how this bacteria would interact with the native soil microbiome and the target plants^109,110^. At the societal scale, questions arise as to the safety and biocontainment strategies needed when releasing engineered organisms, technology access, which intellectual property strategies that can benefit the most people including farmers, and stakeholder analysis to understand if the technology will be adopted.

Biochemical Production—semi-synthetic artemisinin production. Artemisinin is a frontline anti-malarial drug produced in the plant *Artemisia annua*, and its availability can be challenged by seasonal production variation^111^. Microbial bioproduction of more artemisinin requires understanding and engineering across scales. Often bioproduction strategies genetically integrate metabolic pathways into a heterologous host that is then further engineered to make the molecule of interest^18^. At the molecular scale, artemisinin production requires tailored cytochrome P450s and dehydrogenases^96^. At the network scale, these enzymes, along with others, must work together in metabolic pathways with carbon flux carefully controlled to minimize toxic intermediates and side reactions^112,113^. This control requires selection of an appropriate cellular scale host organism that can support the necessary central carbon metabolism and tolerate the acid toxicity of the product^114,115^. As production is scaled, the communities scale becomes important, as scale up requires populations of cells to interact with one another in a complex bioreactor environment where availability and transport of nutrients (e.g., oxygen levels, pH) can become important^116–118^. At the societal scale, questions of cost and profitability, sustainability of production, infrastructure requirements, accessibility to the biochemicals, public perception, and acceptance of the technologies naturally arise.

Human Health—CAR-T cell therapy. Chimeric antigen receptor (CAR) T-cell therapy is a promising approach to provide treatments for an expanding range of cancers^97,98,119^. CAR-T therapies are designed to reprogram the natural abilities of the human immune system to recognize cancer cells and trigger their destruction, and as such they require engineering and consideration across multiple scales. At the molecular scale, a key challenge is designing the CAR protein to recognize features that are unique to the surface of cancer cells while not recognizing healthy cells^120^. Once a cancer cell is recognized, the CAR must activate processes at the network scale within the T cell, triggering cell-mediated killing and gene expression programs^121^. At the cellular scale, the importance of cell identity becomes critical, since CARs can be implemented in a range of immune cell types, with each choice impacting CAR performance^121^. At the biological communities scale, concepts related to side effects (including off-target and on-target activity) become important, creating a natural interface to the molecular scale at which CAR variants can be engineered to have improved specificity^122^. In this scale, concepts such as transport also become important, such as distinct challenges associated with using CAR-T cell therapies to treat solid tumors because of limited penetration, as compared to blood cancers in which T cells can more readily access cancerous cells. At the societal scale, challenges and concepts related to safety, ethics, clinical trials and cost and access of the treatment become important when analyzing the success of the technology^123^

## A case studies-based course in the deconstruction approach

Our course teaches senior undergraduate students and first-year graduate students from a range of degree programs how to analyze problems and solutions related to synthetic biology through the deconstruction approach. The learning objectives of this course are for students to be able to: (1) deconstruct biological phenomena along the scales that they occur; (2) analyze how engineering choices made at one scale affect biological function at another scale; (3) assemble potential synthetic biology solutions to global challenges across scales; and (4) identify the scientific value and impacts of synthetic biology research on broader societal goals, as well as ethical considerations that arise. The course has no prerequisites and was designed to achieve these learning objectives through a case studies pedagogical approach, which is proven to enhance learning and student engagement^88^, allowing integration of multi-disciplinary concepts across scales.

**Fig. 2:**
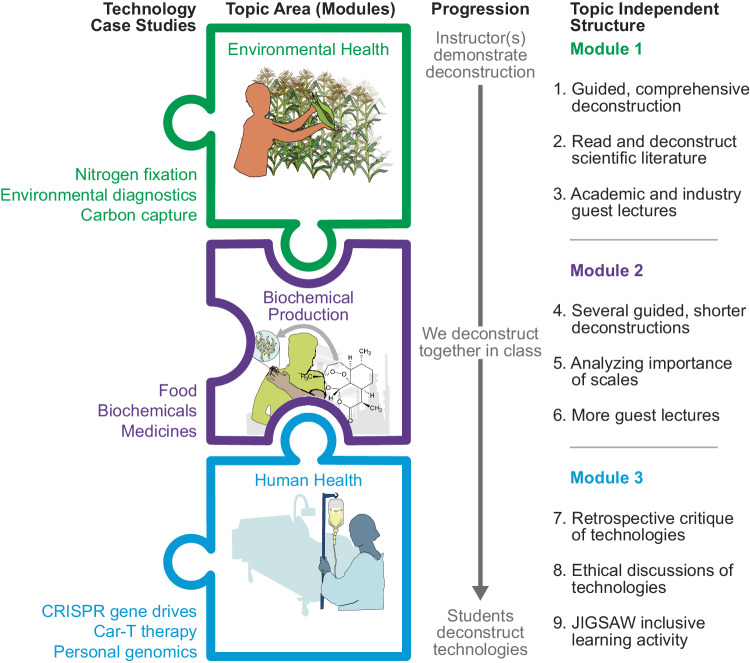
The modular nature of the deconstruction course provides a topic-independent structure for learning. The course is split across three modules with each subsequent module exploring deeper concepts of the deconstruction approach. Different case studies can be used to implement each module, depending on instructor and student interests. Here we show the progression from environmental health to biochemical production to human health topics in the Northwestern course.

For the course, we identified three of the most pressing global challenge areas currently being addressed by synthetic biology to develop case studies—environmental health, biochemical production, and human health (Box 1)^2^. Each challenge area is taught over the course of a three-week module and includes a historical basis for the global challenge (e.g., defining the problem), current synthetic biology research and commercial endeavors in this area, a deconstruction of at least one poignant example, homework assignments (e.g., investigating and designing solutions), student presentations (e.g., explanation), and a guest lecture by an expert in that area. We introduce each challenge area loosely based on the Heilmeier Catechism^89^, defining the problem, how it is addressed today, how synthetic biology might play a role in addressing it, and a discussion on the societal risks, success, and future of synthetic biology in the challenge area. Each module builds on the previous module, adding a deeper layer of understanding of the deconstruction approach (Fig. 2). For example, in the first module we define the scales in the context of a guided case study, in the second module we ask students to weight the importance of each scale to a chosen technology, and in the third module students tackle the challenges at the interfaces between scales. While our course used environmental health for module 1, biochemical production for module 2, and human health for module 3 (Fig. 2), the progression of modules can be taught using any topic sequence, allowing the course to be adapted to the needs or interest of different teaching environments and to new topics that emerge as the field progresses. In addition, the division of the course into modules is naturally amenable to team teaching approaches.

We begin the course by introducing environmental health challenges in the context of United Nations Sustainable Development Goal 3^90^—good health and well-being—and survey the many ways synthetic biology could contribute to solutions in soil, water and air quality, carbon sequestration, waste valorization, remediation, sustainable resource recovery, sustainable biomaterials, recycling, and sustainable fertilizers. We then focus on our first major deconstruction case study on bacterial nitrogen fixation for sustainable fertilizers (Box 1). The nitrogen fixation example also serves as the first introduction to the five scales, as it is deconstructed in the narrative of imagining a synthetic biologist wanting to address the environmental challenge of chemical fertilizers. After a historical introduction to Crooke’s challenge of the need for fertilizers, the geopolitics of fertilizer distribution, and the development of the Haber-Bosch process^91^, we then imagine how a synthetic biologist may partner with nature to create a more sustainable way to produce fertilizer. This naturally starts at the cellular scale by identifying nitrogen fixing bacteria, and quickly dives into the molecular and network/circuit scales on the quest to understand how to engineer the microbe to fix more nitrogen through understanding the nitrogenase enzyme complex and its regulation^92,93^. Reviewing the literature gets us back to the cellular scale to understand which microbes are optimal^94,95^. The biological communities and societal scales naturally emerge when we consider applying engineered microbes to the field. Two guest lectures in this area, one focusing on academic synthetic biology research in this area and another representing synthetic biology startup companies, give students multiple perspectives to understand how this area is actively being pursued.

The focus on fertilizer and agriculture naturally transitions the course to the biochemical production challenge area, where we begin by understanding how commodities such as food, energy, water, materials, and chemicals are intricately linked, and how holistic understanding of a challenge area can give rise to useful solutions. We deconstruct early advances of molecular biology and early synthetic biology technologies such as golden rice, Roundup Ready® crops, and first, second, and third generation biofuels. Our major deconstruction case study in this section is the semi-synthetic artemisinin project^96^ (Box 1), where we use class time to deconstruct the technology along each scale and identify the scales in which key hurdles were overcome during the project. Importantly, we discuss the number of resources that were dedicated to the project, the amount of fundamental knowledge that was gained, the technologies developed during the project that are being used in other areas of synthetic biology, and the current commercial use of the technology as way to evaluate the success of the project. An industry speaker is included in this section to give students perspective on sustainable bioproduction products that are actively being marketed and sold.

The course finishes with the human health challenge area, where we begin by introducing the unique layers of complexity that occur at the biological communities and societal scales. We frame the need for synthetic biology solutions in human health by discussing the historical development of pharmaceuticals and the promise of synthetic biology for developing new therapeutic approaches^6^. We then dive into cell-based therapies and recent synthetic biology tools that allow for molecular, network, and cellular scale engineering of mammalian cells, and control of variability across a population of cells. Our deconstruction case studies in this section are CAR-T-cell therapies^97,98^ (Box 1) and gene drives^99^. Following a student-led deconstruction of these activities, we use discussion-based learning techniques to emphasize the ethics of human subject research through case studies on the use of HeLa cells and personal genomics. Our guest lecturer in this area is a societal scale expert (e.g., bioethicist, artist) that emphasizes the application of societal scale concepts in the course. In addition, we include a guest lecture from one of our faculty to introduce research actively being pursued in our institution.

**Fig. 3:**
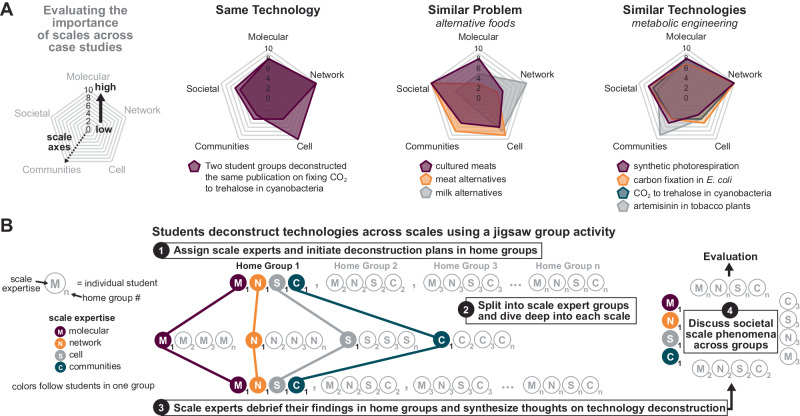
Individual assignments, group activities, and inclusive teaching practices enhance student learning of synthetic biology using the scales approach. **A** Students deconstructed technologies in groups of two and assessed the importance of each scale for their given technology. Each group was asked to rank how important each scale was for their selected synthetic biology technology from 0 (no importance) to 10 (high importance). Radar plots are displayed for different student groups’ responses where each geometric shape or area represents one response. Differences in student responses on ‘the importance of scales’ are depicted in three ways: deconstructing the same technology, deconstructing different technologies that aim to tackle a similar problem, and deconstructing similar technologies within a research area. **B** Students deconstructed technologies across scales using an inclusive teaching technique called a jigsaw group activity. Each circle represents one student in the course, each letter is a specific scale, and each number corresponds to a specific grouping of students that are assigned a different technology. Home groups allow students to frame their deconstruction across different scales, while scale expert groups allow students to gain expertise in a scale by comparing across different technologies. Reassembling back into home groups allows students to share their expertise and learn from each other. Discussing the societal scale across technologies as a class allows comparisons between different technologies.

An important component of our pedagogy is activities for students to actively deconstruct technologies across scales, including individual assignments, small-group evaluation of technologies, and cooperative learning activities based on inclusive teaching practices^100–102^ (Fig. 3). This begins in the environmental health section where students are assigned to pick a technology and deconstruct it *without* the scales framework introduced (*assignment 1*). Once the nitrogen fixation technology is deconstructed in lectures, they are then asked to revisit the deconstruction of this same technology with the scales framework (*assignment 2*), and present to class. In the biochemical production section, the course begins to flip from instructor-centric to student-centric deconstructions through additional group work. We randomly paired students together and asked them to pick a technology to deconstruct and go beyond just identifying the scales by weighting the importance of each scale within their chosen technology (Fig. 3A). We found that students interpret the importance of scales differently. For example, two students focusing on food alternatives found different scales are important for different technologies, while in some metabolic engineering examples, students found the network/circuit scale to be of importance regardless of the selected technology. This type of cross-case comparison helped promote the abstraction of deconstruction concepts.

In the human health section, CAR-T and gene drives are deconstructed through a unique jigsaw method, a cooperative and inclusive learning approach that requires students to address a complex problem from various theoretical and/or methodological approaches (Fig. 3B)^100–102^. Students are first split into several “home (jigsaw) groups” consisting of one “scale expert” at the molecular, network, cell/cell-free, and biological communities scales to discuss a game plan to deconstruct their assigned technology. While students do not necessarily have expertise in their assigned scale, using the term ‘expert’ is meant to inspire confidence in students to learn scale concepts and then empower them to teach their peers. Students then divide small “scale expert groups” and use peer instruction to develop deep knowledge in a specific scale (a ‘piece of the puzzle’). The students then return to their home groups, synthesize their expert information into a compelling deconstruction of their technology and together discuss the societal scale. At the end of this activity, we come back to a large group discussion of technology challenges across scale interfaces and the societal implications of the technology.

Throughout the course, each student is assigned to conduct a newsreel presentation by presenting one synthetic biology research article and one news item of their choice to the class using the scales framework, creating a consistent source for ethics discussions and other societal scale topics. Finally, students perform and present a deep dive deconstruction of a technology of their choice as their final project. In this way the course incorporates a wide range of technology case studies that are both instructor and student chosen. The ability for students to drive most of the topic selection (e.g., engaging in the practice of science) in this course builds off the known positive impact of choice on student engagement^103^ and allows course content to adapt as the field of synthetic biology evolves.

By framing the course around biotechnologies and the scales of synthetic biology, we can teach synthetic biology in a way that is agnostic to student backgrounds and expertise. In this way, we can introduce multi-disciplinary concepts from biology, chemistry, physics, mathematics, computer science, engineering, and the social sciences in the context that they are needed within a given scale. This helps students identify where their background and expertise can be incorporated within a synthetic biology technology. The scales framing also allows students to identify their own knowledge gaps so that they can fill them with further study and collaboration.

## Evaluating success of the deconstruction approach

Teaching a course rooted in quantitative fundamentals of synthetic biology technologies, but largely taught through learning how to define problems, develop models, construct explanations, and build arguments (e.g., scientific practices) has proven to be a rewarding experience for students. In total, 103 students from chemistry, biology, biomedical engineering, civil and environmental engineering, and chemical engineering programs took the course across three separate years that the course was offered at Northwestern University. Students across implementations of the course resonated with the deconstruction approach as can be seen from an analysis of end-of-course written reflections as part of their final projects (Table 1). Responses, subjected to thematic analysis^104^, revealed that students not only enjoyed the course but also developed holistic ways of thinking, critical thinking skills, an ability to recognize challenges at the interface between scales, and an understanding of how they would use the deconstruction approach outside the course (e.g., reading literature, career aspirations). Years after taking the course, one student reflected in an interview that, “[the scales framework] has been super helpful for the conception of my own research because I’m often on the lower scales, more of the mechanisms and specific interactions of molecules and proteins. Anytime we’re making single changes to add more of this one component to our mixture, it really changes everything else … and it goes beyond these lower-level interactions. It’s not that I’m consciously trying to think in that way, but I think it’s been baked into me. These scales all do interact and are relevant. Even when it feels like I’m making small changes, I feel I need to stop and consider the potential for repercussions and effects that would climb up the ladder.” Students have applied and seen value in the skills developed in the course years after taking the course.

**Table 1:** Thematic analysis of final assignment reflections

Statement category	Example quote	Respondents (*n* = 80)	
New type of thinking (holistic/systemic)	*I am now able to gain a holistic understanding of synthetic biology technologies by deconstructing them* via *the “scales” framework*.	39%	31
Recognize challenges at the interface between scales	*I have often found that when I get confused, it is usually because there is a scale interface challenge that I need to further deconstruct*.	39%	31
Enthusiasm/enjoyment	*It has made my scientific journey more enjoyable*.	35%	28
Problem-solving/critical thinking/problem-identification	*I am now able to identify how challenges at the interfaces of these scales affect the overall technology, as well as what strategies might be needed to resolve them*.	34%	27
Importance of societal scale &/or ethical considerations	*Now I know that not only is the societal scale important, but it interfaces closely and crucially with each and every other scale, and that not including societal impact in the development of a new technology is a mistake*.	34%	27
Generic growth (i.e., better understanding of scales)	*I went back and read my first assignment, and compared it to now, I could clearly see an improvement in the depth of my understanding of the scales framework*.	33%	26
Helps facilitate reading syn bio papers/research	*Reading research articles have become much less cumbersome*.	31%	25
Application to career/future education	*I am sure I will apply what I learnt in this class throughout the rest of my time in academia as well in other aspects of life*.	29%	23
Syn bio is multi-disciplinary & collaborative	*I have come to realize the immense collaboration that occurs within the synthetic biology field. The vast range of knowledge needed for creating one technology just shows how more research and development can occur when people of diverse backgrounds come together*.	18%	14
Learning about/focusing on less familiar scales	*I gained a lot more confidence with the smaller scales, which are not typically the focus of my discipline*.	15%	12
Learning about/understanding technologies	*[I learned] how using CRISPR Cas9 systems can enable the large-scale production of silk spidroins*.	14%	11
Scales overlap/are not clearly delineated	*One thing I struggled with is that not everything fits super easily into one or even two scales*.	8%	6

Respondents across three implementations of the course (80 total respondents; 23 students did not respond) were asked to reflect on their final projects. Specifically, students were asked “Compared to how you started at the beginning of the course, what do you now know about deconstructing synthetic biology solutions ‘along scales’? How has your thinking changed/developed?”. Using thematic analysis, answers were categorized by theme; the number of responses that fell into each category are shown.

## Integrating the societal scale into a STEM course

An important goal of the deconstruction approach is to train students to think about the societal scale impacts of their work as it is being conceptualized, rather than after it has been done. Traditional science and engineering training often leaves out societal scale components or relegates them to special courses in the humanities (e.g., bioethics) or business (e.g., intellectual property) that do not fully integrate these topics within science and engineering. We integrated the societal scale into our course in three specific ways: (1) training students to identify challenges at the societal scale, and biological functions needed to address these challenges, through course assignments; (2) creating space for students to explore the connectedness of how science and engineering choices made at one scale could drive outcomes at the societal scale through in-class discussion grounded in bioethics best practices^105^; and (3) inviting a guest lecturer with expertise in bioethics and the societal scale to guide an informed and meaningful discussion around this scale using examples from their own work. Our intent was to introduce students to the many topics this scale encompasses (e.g., bioethics, technology access and equity, intellectual property, business models, investment strategies, and policy), teach them to identify connections between the societal scale and the four other scales and teach them how to discuss and grapple with societal scale challenges for any technology.

Our specific societal scale and bioethics discussion activities were based on bioethics best practices^105^. We conducted think-pair-share class discussions with prompts along several themes: (1) themes related to societal perceptions of biotechnology; (2) themes related to unintended consequences of developing biotechnologies; and (3) themes related to additional safeguards and regulatory processes that could be developed in response to unintended consequences. For example, during the human health part of the course when we discussed gene drives as a method to combat malaria. Our discussions touched on intellectual property, genetically modified organisms, and regulations; molecular and cellular approaches to biocontainment to mitigate risk; and public perception of technology and what is natural. We wove these types of concepts into each case study, student deconstruction assignments and discussions, and a standalone discussion of the ethics of human subject research. The most recent iteration of the course also had an artist lead discussion of how science and art can interface to impact the world. As a result, students often expressed excitement and eagerness to think about the societal scale and how they might advance or disrupt the world in which we live. In our discussions we did not try to seek an answer to questions at this scale but rather focused on presenting and discussing different viewpoints, emphasizing the importance of considering societal scale challenges. Many students came away with their viewpoints expanded, with 34% commenting on the importance of societal scale thinking (Table 1).

## Adapting the approach to other learning environments

In developing the course, we created a syllabus, a schedule, and content that is designed to be adapted to other learning environments. Our goal is for the scales framework and the deconstruction approach to be adaptable to support a range of learning objectives within different institutions and programs and to be adapted to changes with the field. Towards this goal, we have created and included here a modular version of our course structure, a syllabus, and the three evaluated deconstruction assignments with corresponding rubrics for any instructor who would like to use them or adapt them for a course in synthetic biology (see Supporting Information). The content can be used in several ways. If instructors are comfortable with the progression of topics from environmental health to biochemical production to human health, then the course plan could be used verbatim to implement a full course that could serve as an introduction to synthetic biology, or as a second course in synthetic biology. If instructors would rather begin with a different topic area, then they could use our course plan and structure as an example and choose a different framing example in a different topic area (Box 1) to do a full deconstruction of a technology at the beginning of the course, followed by similar activities to explore other topic areas. This method could also be used to implement a standalone module on the deconstruction approach within a different synthetic biology course. In this model, case studies can be used to get students excited by the field before deep diving into synthetic biology tools and principles that are typically discussed in introductory synthetic biology courses. It was important to select case studies that we as instructors had expertise in to give the most enriching experience for our students and to help facilitate their learning. Including more formal cross-case study comparisons would help enhance student understanding of the deconstruction approach and mobilize knowledge. Portions of the course could even be used as modules to add an ethics component to an existing synthetic biology course. In addition, the three framing deconstruction assignments can be added into existing courses to teach and evaluate student learning of the deconstruction approach. While our implementation of the course was tailored to a mixed class of advanced undergraduates, masters, and beginning PhD students, we envision the approach being easily tailored to other groups.

Over three years of implementing this course, several best practices for implementation appeared. Initially the course was developed for synchronous, remote learning and was adapted to in-person sessions which means that the course is fully compatible with remote, in-person, or hybrid teaching. At the heart of the course are student presentations and discussions. This made the course challenging to implement when class sizes reached more than 30 students. The number and type of presentations can be changed to address this. We also struggled to identify the proper number of assignments and in-class activities given that most assignments were free-form writing. Giving comprehensive rubrics and instructions helped manage expectations and improved student enjoyment of the course. While we had no prerequisites for the course, many students who took previous biology and/or synthetic biology courses had an advantage. Implementations of the course where this is the only available course in synthetic biology may benefit from an “introduction to synthetic biology” module to familiarize students with tools and techniques in the field. Despite differences in prior knowledge, we had students come to this course from chemistry, biology, engineering, and biotechnology and left inspired to work in synthetic biology.

## Looking to the future

As the field of synthetic biology matures, there is a compelling opportunity to explore common training approaches across institutions that can be used to accelerate progress in the field even further. As a highly multi-disciplinary field, it can be challenging to find a convergent training approach that incorporates cross-field concepts while giving students and practitioners a common language to integrate these concepts towards a common engineering goal. We believe that by emphasizing the scales of engineered biological systems and their application use cases, the scales framework and the deconstruction approach helps to achieve this goal and can incorporate discipline-specific concepts simultaneously. In this way, the scales framework facilitates the teaching of “science practices” (e.g., modeling, explanation, argumentation)^25^ and core ideas of 21st-century science which will facilitate developing disciplinary expertise and versatility^26,27^.

Here, the scales approach has allowed us to train students from a range of disciplinary backgrounds in common, multi-disciplinary concepts. Teaching students first how to deconstruct technologies along scales and then identify concepts that apply at each scale, allows them to integrate diverse concepts together in the context of how they are used for engineering. While biological emergent behavior lends itself to the scales-based framework, synthetic biology has traditionally been skewed towards the molecular and circuits/network scales. In contrast, bioengineering and biomedical engineering are traditionally skewed towards the cell and biological communities scales. Yet often the goals of synthetic biologists and bio/biomedical engineers are the same: to tackle a global challenge with biological solutions. The scales framework allows for appreciation of all the scales, which we hope encourages researchers to seek out knowledge of traditionally overlooked scales and work across scales to develop impactful biotechnologies.

While we have started to lay the framework for a deconstruction approach to teaching synthetic biology, it is far from complete. As the field evolves, it is our hope that the deconstruction approach evolves with it. We can already see evidence of this through the definition of the scales. For example, in our recent implementation of the course during a deep dive into CRISPR gene drives, students challenged our definition of the biological communities scale and actively discussed whether a new scale should be added to encapsulate concepts relevant to organismal populations such as population genetics. In addition, drawing connections to how different other fields use the scales framework—like computer engineering where technologies are built from transistors, to circuits, to chips, to devices—can further refine its application to synthetic biology and drive additional innovation. For example, the existence of computer-aided design tools that can be used within and across scales to design computer systems is a powerful encapsulation of the scales framework and is a particularly exciting prospect for synthetic biology^106,107^. Using this central framework, iterations of this course could be developed that bring in additional discipline-specific concepts, pointing out when in each synthetic biology technology those concepts can be applied. In this way, a student trained in that discipline can learn when and how to collaborate with researchers in other disciplines, addressing the need to learn to integrate and traverse disciplines. We anticipate that continued adoption, discussion, and development of the deconstruction approach will allow the concepts to be refined to match the needs of the field.

We envision the deconstruction approach to be more than just a pedagogical approach to teaching synthetic biology. Rather, we hope that it is viewed as a way of thinking for synthetic biologists of the future. By teaching students to think across scales, we hope that their holistic view of what it takes to make a successful synthetic biology technology will allow them to identify knowledge gaps that can be filled by new learning, new collaborations, or even drive new research to fill those gaps. By placing the societal scale on equal footing with the other scales, we hope to create an ethically minded workforce that will drive responsible innovation. And by emphasizing how many disciplines are needed across scales to achieve success, we hope to welcome diverse perspectives to the field of synthetic biology so we can all work towards solving society’s grand challenges together.

### Supporting Information

The following supplemental materials are provided on the Northwestern Arch database (10.21985/n2-x989-tb47) to aid the adoption and adaption of the scales framework and deconstruction approach to other learning environments:

Northwestern_CSB_Deconstructing_SynBio_Content_Map.pdf – a table outlining how the course content can be delivered across a ten-week course. Modules on environmental health, biochemical production and human health are outlined. A schedule for the provided assignments is given, along with how to integrate guest lectures.

Northwestern_CSB_Deconstructing_SynBio_Syllabus.pdf – example syllabus for the deconstructing synthetic biology course.

Northwestern_CSB_Deconstructing_SynBio_Assignment_1-First_Deconstruction.pdf – the first deconstruction assignment given to students before they have been taught about the scales framework.

Northwestern_CSB_Deconstructing_SynBio_Assignment_2-Second_Deconstruction.pdf – the second deconstruction assignment given to students immediately after they have been taught about the scales framework.

Northwestern_CSB_Deconstructing_SynBio_Assignment_3-Final_Project.pdf – the course final project entailing a deep dive deconstruction using all the principles learned in the course.

## Data Availability

The full set of deidentified responses used for thematic analysis in Table 1 can be made available upon reasonable request pending ethical consideration of intended use.
